# A novel role for P2X7 receptor signalling in the survival of mouse embryonic stem cells^☆^^☆☆^

**DOI:** 10.1016/j.cellsig.2011.11.012

**Published:** 2012-03

**Authors:** Belinda A.N. Thompson, Michael P. Storm, James Hewinson, Sarah Hogg, Melanie J. Welham, Amanda B. MacKenzie

**Affiliations:** a Department of Pharmacy and Pharmacology, University of Bath, Bath, BA2 7AY, UK; b Centre for Regenerative Medicine, University of Bath, Bath, BA2 7AY, UK; c School of Physiology and Pharmacology, University of Bristol, UK

**Keywords:** ES, embryonic stem, m, mouse, HEK293, human embryonic kidney 293, Ion channel, ATP, Stem cell, Receptor, Survival, P2X receptor

## Abstract

The growth of a pluripotent embryonic stem (ES) cell population is dependent on cell survival, proliferation and self-renewal. The nucleotide ATP represents an important extracellular signalling molecule that regulates the survival of differentiated cells, however, its role is largely undefined in embryonic stem cells. Here we report a role for ATP-gated P2X7 receptors in ES cell survival. The functional expression of P2X7 receptors in undifferentiated mouse ES cells is demonstrated using a selective P2X7 antagonist and small interfering RNA knockdown of these receptors. Our data illustrate a key role for the P2X7 receptor as an essential pro-survival signal required for optimal ES cell colony growth in the presence of leukemia inhibitor factor (LIF). However, chronic exposure to exogenous ATP leads to rapid P2X7-dependent cell death via necrosis. Together, these data demonstrate a novel role for P2X7 receptors in regulation of ES cell behaviour where they can mediate either a pro-survival or pro-death signal depending on the mode of activation.

## Highlights

► Expression of functional ATP-gated P2X7 receptors in mouse embryonic stem cells. ► Multiple P2X7 variants detected by RT-PCR. ► Stem cell P2X7 couples to ion channel opening and a dye uptake pathway. ► P2X7 receptor can function as either a pro-survival or cytotoxic receptor.

## 1 Introduction

Mouse embryonic stem (ES) cells are derived from the inner cell mass of day 3.5 pre-implantation blastocysts [1,2]. The unique and defining properties of ES cells are that they can self-renew, proliferate whilst remaining in an undifferentiated state and are pluripotent, maintaining the capacity to differentiate into all cell types of the adult organism [3]. ES cell behaviour is regulated by external signalling molecules including LIF, via STAT3 and Myc activation [4–6]; bone morphogenetic proteins (BMP2 and 4) via the induction of Id proteins [7] and Wnts [8]. Extracellular adenosine 5′ triphosphate (ATP) has been extensively studied as an autocrine- and paracrine-signalling molecule in differentiated cells of the adult organism [9]. Extracellular ATP has been shown to act as both a pro-survival signal in a range of cancers and a pro-death signal in the innate immune system [10]. However, relatively little is known about the potential contribution of ATP signalling during embryonic development or whether it influences the behaviour of ES cells. Extracellular ATP binds to membrane purinergic receptors that include ionotropic P2X receptors and metabotropic P2Y receptors. An early study reported the expression of P2X3, P2X4, P2Y1 and P2Y2 receptor mRNA in mouse ES cells (E14TG2a) where ATP is reported to have a proliferative action [11]. However, this study did not investigate the expression or contribution of P2X7 receptors [11]. Whilst other studies report mRNA encoding P2X2, P2X5, P2X7, P2Y1, P2Y2 and P2Y6 can be detected in mouse ES cells [12,13], the functional expression and potential role of P2X7 receptor-coupled ion channels in undifferentiated mouse ES cells have not been investigated.

P2X7 is a unique member of the P2X family since: i) relatively high concentrations of ATP are required to fully activate the receptor, and ii) the receptor has an extended C-terminal, intracellular domain, which is involved in coupling receptor activation to downstream signalling events such as membrane permeabilisation to large molecular dyes [14]. These signalling events are often associated with cell death, and sustained stimulation with millimolar ATP leads to apoptotic or necrotic cell death [10]. Indeed, in the immune system ATP is described as a ‘danger associated molecular pattern (DAMP)’ where it signals as a pro-apoptotic/necrotic molecule. However, several studies have reported a pro-survival role of P2X7 receptors [15–19]. It is proposed that P2X7 receptor can detect low basal levels of ATP, likely to be due to autocrine or paracrine ATP release, that promotes cell survival and growth. Indeed, increased P2X7 expression is reported as a cancer cell biomarker where the receptor couples to a change in growth rate and cell survival [19]. Mitochondria calcium (Ca^2+^), endoplasmic reticulum Ca^2 +^ and the transcription factor NFATc have all been implicated in P2X7-driven cell survival [16,17]. Overall, P2X7 receptors play an important role in the survival of specific cells in the adult organism.

This study has specifically focused on the functional expression of the P2X7 receptor which is classically considered to be a receptor expressed by immune and epithelial cells of the adult organism. Specifically, we have investigated the expression of specific P2X7 receptor isoforms by RT-PCR and the functional expression of P2X7 gated ion channels. Using pharmacological inhibition and knock-down approaches to inhibit P2X7 receptor activation, we reveal the role of P2X7 in mouse ES cell survival. The outcomes of this study have important implications in our understanding of ion channel expression and extracellular signalling molecules in mouse ES cell function.

## 2 Materials and methods

### 2.1 Reagents

Reagents were obtained from Sigma-Aldrich Company Ltd., Dorset, UK unless stated. Tissue culture reagents were obtained from Invitrogen, Paisley, UK, unless otherwise stated. A438079 was purchased from Tocris, Bristol, UK. Penicillin/streptomycin was from Invitrogen and endothelial cell growth medium (ECGM) from PromoCell (Heidelberg, Germany).

### 2.2 ES cell culture

The mouse ES cell line E14tg2a was maintained as previously described [20,21] by culture in knock-out Dulbecco's Modified Eagle Medium (KO-DMEM) (Invitrogen) supplemented with 15% (v/v) knock-out serum replacement, 2 mM glutamine, 0.1 mM 2-mercaptoethanol, 1% (v/v) non-essential amino acids and 1000 U/ml ESGRO (LIF) (Chemicon Temecula, CA) (complete media). Cells were washed in phosphate buffered saline and detached with 0.05% trypsin/EDTA before being plated as required.

### 2.3 HUVEC cell culture

The use of human umbilical cords and isolated cells in this research was granted ethical consent (Bath Local Research Committee reference BA635). Endothelial cells were isolated from umbilical cords and cultured using a modified protocol previously described [22]. Briefly, umbilical veins were cannulated, perfused with HBSS to remove cord blood and then incubated with collagenase (1 mg ml^− 1^) for 45 min at ambient room temperature (20–25 °C) to disrupt the endothelial lining. Following incubation the collagenase solution was washed through with HBSS and the cell suspension was collected. Cells were collected by centrifugation at 240 ×*g* for 4 min and re-suspended in ECGM supplemented with 100 U ml^− 1^ penicillin and 100 μg ml^− 1^ streptomycin. HUVECs were grown on tissue culture treated plastics and medium was changed three times a week. Upon reaching confluence cells were dissociated using a non-enzymatic cell dissociation solution (C5789, Sigma, UK). In all experiments HUVECs were used at passage 2 and measurements performed in a physiological saline containing (in mM) 130 NaCl, 5 KCl, 1.5 CaCl_2_, 1 MgCl_2_, 5 NaHCO_3_, 1.5 KH_2_PO_4_, 10 d-glucose and 25 HEPES (pH 7.3 with NaOH).

### 2.4 RT-PCR

RNA was extracted by lysis in 200 μl TRIzol reagent (Invitrogen) according to manufacturer protocols, RNA was isolated, DNase treated and reverse transcribed as previously described [23]. RT-PCR was carried out using HotStarTaq Plus DNA polymerase (Qiagen, Hilden, Germany) according to the manufacturer recommendations using the primers listed in Table 1.

### 2.5 siRNA transfections

ON-TARGET*plus* SmartPOOL siRNA targeting murine P2X7 mRNA and a non-targeting (NT) control were purchased from Dharmacon (Lafayette, CO, USA) and transfected at a range of concentrations as previously described [13]. Lipofectamine 2000 (Invitrogen) and siRNA were incubated separately with 50 μl KO-DMEM, without supplements for 5 min, these were gently mixed and incubated for a further 20 min at room temperature. 1 × 10^5^ ES cells were then added to the siRNA/Lipofectamine mix and plated onto a gelatin-coated 12 well tray (NUNC) in complete media. 24 h later media was replaced and 48 h after initial transfection cells were washed in PBS and transfected in a similar fashion whilst still attached. After a further 24 h transfected cells were washed, trypsinised and plated for ethidium (Et^+^) influx assays as well as RNA extraction to evaluate mRNA reduction. For each experiment, pan-P2X7 primers were used to detect the expression of P2X7.

### 2.6 Ethidium influx

Measurements of Et^+^ influx, as a measure of pore formation, are as previously described [24–26]. Prior to Et^+^ influx measurement, cells were plated for 4 h in complete medium in a gelatin-coated 96 well black plate at a seeding density of 1.5 × 10^6^ ml^− 1^. Complete medium was removed and cells were incubated at 37 °C in PBS containing 25 μM EtBr. Fluorescent measurements were performed using a multi-detection plate reader (Fluostar Optima, BMG Labtech, UK) with excitation wavelength of 540 nm and emission wavelength of 590 nm. For the ATP dose response curve, the rates of dye influx (dye uptake slopes) were normalised to 1 mM ATP responses.

### 2.7 Whole cell patch clamp recordings

For whole cell recordings, mouse ES cells were plated onto gelatin-coated glass cover slips for 4 h and used within 4 h. Whole cell recordings were performed as previously described [27] using a HEKA EPC10 patch clamp amplifier and data collected using PatchMaster software (HEKA). Current recordings were performed at ambient room temperature. Membrane potential was held at − 60 mV. Agonists and antagonists were applied using a rapid solution changer (Biologic, France). Borosilicate glass microelectrodes, 3–5 MΩ are filled with solutions containing (in mM) 145 KCl, 10 HEPES and 1 EGTA (pH 7.3 with KOH). Cell stimulations were carried out using an external physiological salt solution containing (in mM) 147 NaCl, 2 KCl, 10 HEPES, 12 glucose, 2 CaCl_2_ and 1 MgCl_2_ (pH 7.3 with NaOH). Peak currents were defined as the maximal amplitude of response during the agonist application in the presence or absence of antagonists; responses were plotted as current density (pA/pF).

### 2.8 Alkaline phosphatase self-renewal assays

E14tg2a mouse ES cells were plated at a cell density of 1500 cells/well in a gelatin-coated six-well dish (Nunc) containing KO-DMEM medium, serum replacement, 10% (v/v) foetal bovine serum (Hyclone, ThermoFisher) and 1000 U.ml^− 1^ LIF. Alternatively GMEM + 10% (v/v) foetal bovine serum (Hyclone, ThermoFisher) + 1000 U.ml^− 1^ LIF were used. A438079 was added as indicated. After culture for 5 days, ES cells were stained for alkaline phosphatase activity as previously described (20) by incubating for 25 min in 0.1 M Tris solution (pH 9.2) containing 0.2 mg.ml^− 1^ Napthol AS-MX phosphate and 0.5 mg.ml^− 1^ Fast Violet B salt. Colony numbers and percentage alkaline phosphatase positive cells were counted.

### 2.9 Proliferation assay

E14tg2a mouse ES cells were cultured on gelatin-coated Nunc tissue culture plastic in KO-DMEM medium with knock-out serum replacement with the addition of 1000 U.ml^− 1^ LIF. For proliferation assays, 3000 cells/well were plated into gelatin-coated 96 well plates in GMEM culture medium with 10% Hyclone FBS and 1000 U.ml^− 1^ LIF in the presence of inhibitors as indicated. Proliferation was evaluated using a CellTitre 96 AQueous (MTS) assays (Promega) where absorbance (490 nm) was measured using a VersaMax plate reader (Molecular Devices).

### 2.10 Online ATP assay

A sensitive bioluminescent-based assay capable of detecting ATP in the range of 2 × 10^− 12^ to 8 × 10^− 5^ M was used to analyse ATP hydrolysis by HUVECs and ES cells. The ATP assay mix (containing luciferase, luciferin, MgSO_4_, dithiothreitol, EDTA, bovine serum albumin, and tricine buffer salts) used in this procedure was prepared by adding 5 ml sterile distilled water to the lyophilised powder supplied and 100 μl aliquots of this concentrate were stored at − 20 °C. On-line ATP assays were performed using a FLUOstar OPTIMA multi-well plate reader (BMG Labtech, Offenburg, Germany) with temperature control set at 30 °C to prevent luciferase inactivation that occurs above this temperature [28]. HUVECs were seeded 45,000 cells per well in white, 96-well, tissue culture-treated plates the day before experimentation. E14tg2a mouse ES cells were cultured on gelatin coated Nunc tissue culture plastic (150,000 cells per well) in KO-DMEM medium with knockout serum replacement with the addition of 1000 U.ml^− 1^ LIF. For ATP hydrolysis studies cells were washed once in 100 μl physiological saline to remove culture medium. Measurements of ATP hydrolysis were performed where the luciferin/luciferase mix was added directly onto the cells (diluted 25 times in the final well volume) and luminescence detected as photons by a photomultiplier tube in the plate reader. The rate of ATP degradation is calculated as the change in detected ATP levels per minute in the presence of exogenous ATP.

### 2.11 Cell viability

E14tg2a mouse ES cells were plated for 4 h in complete medium in a gelatin-coated 96 well clear plate at a seeding density of 1.5 × 10^6^ ml^− 1^. Complete medium was removed and cells were incubated at 37 °C in PBS containing the test substance. Supernatants were removed and added to Cytotoxicity Detection Kit (Roche Applied Science, Burgess Hill, UK) reaction mixture. Absorbance of samples at 490 nm was measured using a multi-detection plate reader (Fluostar Optima, BMG Labtech, UK) or a VersaMax plate reader (Molecular Devices). Total cellular LDH was determined by the addition of 1% Triton-X 100 to untreated cells, and the amount of LDH released is expressed as a percentage of the total LDH release.

### 2.12 Nanog-GFP

Nanog-GFP cells (generated by recombineering an eGFP knock-in at a Nanog allele) [29] were cultured on gelatin coated 6-well Nunc dishes at 8 × 10^4^ per dish for 48 h or 3 × 10^4^ for 72 h in KO-DMEM + 15% KO-serum replacement and 1000 U.ml^− 1^ LIF as described above, in the presence or absence of 10 μM A438079. After 48 h or 72 h cells were removed from the dish by trypsin treatment as described above, washed 3× in PBS + 5% FBS and re-suspended in PBS + 5% FBS. Flow cytometry was carried out using a FACSCanto (Becton Dikinson, Oxford, UK) and data was analysed using FACSDiva software. Dead cells were excluded from analysis based on forward and side scatter parameters.

### 2.13 Data analysis

Data points shown in graphs represent the mean ± standard error of the mean (SEM) with a minimum of three independent data points where *n* values are indicated in the figures and text. Where indicated in the figure legend, data were analysed using an unpaired two-tailed t test using GraphPad InStat 3.1 (GraphPad Software). The agonist–dose response curves were fit using *R*/*R*_max_ = 100([*A*]^*n*^_H_ / ([*A*]^*n*^_H_ + *EC*_50_^*n*^_H_), where [*A*] represents agonist concentration, *R* is the fluorescence response induced by [*A*] expressed as a function of the peak maximal agonist response (*R*_max_), *n*_H_ is the Hill coefficient and *EC*_50_ the half maximal response. The pEC_50_ value represents the negative logarithm to base 10 of the *EC*_*50*_ value.

## 3 Results

### 3.1 Expression of functional P2X7 receptors in mouse ES cells

**Fig. 1:**
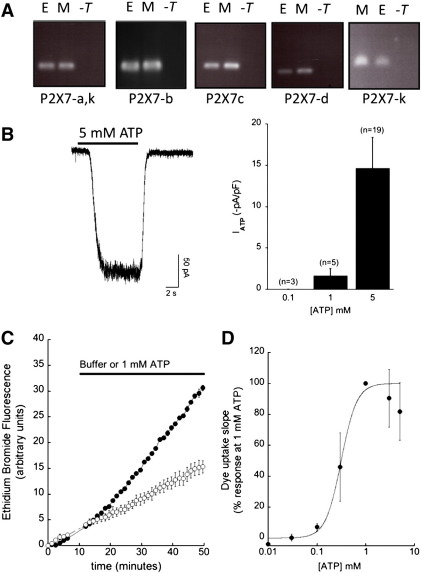
Expression of P2X7 mRNA transcripts and functional assays of P2X receptor expression. (A) RT-PCR was performed for P2X7 variants using the primers described in Table 1. Amplification products of the expected size were detected where E represents mouse embryonic stem cells, M represents J774.2 mouse macrophages and -T is the no template control. (B) Whole cell patch clamp recordings from undifferentiated mouse ES cells at the holding potential of − 60 mV. Histogram summarises the mean peak currents normalised to membrane capacitance detected at the ATP concentrations given. (C) Membrane permeabilisation measured by the uptake of ethidium and corresponding increase in fluorescent signal (544 nm excitation; 590 nm emission). An example kinetic trace of ethidium measurements from mouse ES cells with a saline vehicle (○) versus the addition of 1 mM ATP (●). (D) Concentration response relationship for ATP versus the rate of ethidium influx normalised to 1 mM ATP responses (*n* = 5) as described in methods. Data are plotted as the mean ± SEM.

To date, one study has investigated the functional role of ATP activated P2 receptors in mouse ES cells [11]. The authors report a role for P2Y and P2X in ATP-induced proliferation using broad-spectrum, non-selective P2 receptor inhibitors (suramin and reactive blue 2). This study did not investigate P2X7 mRNA expression though it did detect the expression of P2X3 and P2X4 mRNA. We have investigated the expression of mouse P2X7 splice variants and detect the expression of P2X7a-d and P2X7k by RT-PCR (Fig. 1A). We conducted whole-cell patch clamp recordings to investigate the potential expression of native ATP-gated P2X7 receptors in mouse ES cells. The membrane capacitance of patch-clamped ES cells ranged from 5.9 to 100 pF (21 ± 4.5 pF, *n* = 23). Cells were voltage-clamped at − 60 mV and brief 10 s applications of 5 mM ATP elicited sustained inward currents with a mean current density of 14.6 ± 3.7 pA/pF (*n* = 19, Fig. 1B). Maximal current was detected at 5 mM ATP and no currents could be detected with the application of 100 μM ATP (Fig. 1B). A distinct property of P2X7 activation is the ability to elicit the influx of large molecular weight cations such as the fluorescent dye Et^+^. In mouse ES cells, extracellular ATP triggers Et^+^ uptake with an apparent pEC_50_ value of 3.45 ± 0.13 (*n* = 5) where the maximal rate was detected at 1 mM ATP (Fig. 1C and D). The ATP concentration response curve decreases at ATP concentrations greater than 1 mM ATP, this has also been observed for P2X7 responses in mouse macrophages [24,30].

**Fig. 2:**
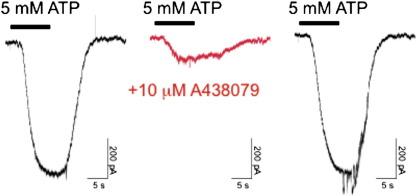
Pharmacological evidence for the expression of P2X7 receptors in mouse ES cells. Whole cell voltage clamp recordings from mouse ES cells with a holding potential of − 60 mV. Reversible inhibition of ATP-mediated current with a selective P2X7 antagonist A438079. Application of 5 mM ATP in the absence or presence of 10 μM A438079 as indicated.

**Fig. 3:**
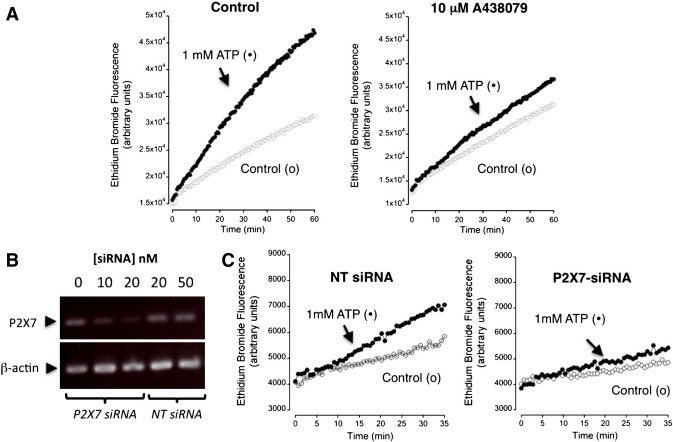
Evidence for P2X7 receptor expression by siRNA knockdown of receptor expression. (A) An example of ATP-mediated ethidium uptake in the absence or presence of 10 μM A438079 as indicated in the figure. Buffer control (ο) or addition of 1 mM ATP (●) as indicated. Representative of 3 independent experiments. (B) Knockdown of P2X7 expression by transfection of mouse ES cells with P2X7 targeting siRNA (P2X7 siRNA) versus a non-targeting control siRNA (NT siRNA) as indicated. P2X7 receptor expression was assessed by semi-quantitative RT-PCR using panP2X7 primers and β-actin as a positive control (primers Table 1). (C) Ethidium uptake is reduced in cells treated with 20 nM siRNA knockdown of P2X7 expression versus cells treated with the 20 nM control non-targeting (NT) siRNA as indicated in the figure. Representative of 3 independent experiments.

We used a P2X7-selective antagonist A438079 [31] and siRNA-knockdown of P2X7 to investigate the contribution of P2X7 to ATP-mediated ionic currents and Et^+^ influx. Co-application of 10 μM A438079 with 5 mM ATP reversibly inhibited evoked currents by 83% (*n* = 3, Fig. 2). The action of A438079 and P2X7-targeted siRNA was investigated on the observed ATP-mediated Et^+^ influx. Co-application of 10 μM A438079 with 1 mM ATP inhibited Et^+^ efflux compared to control rates (*n* = 3, Fig. 3A). Semi-quantitative RT-PCR analysis of ES cells transfected with P2X7-targeting siRNAs showed reduced expression of P2X7 mRNA that was not seen with control non-targeting siRNAs (Fig. 3B). Et^+^ uptake mediated by 1 mM ATP was reduced in ES cells transfected with P2X7 siRNA compared to control non-targeting siRNA (*n* = 3, Fig. 3C). Together, these data demonstrate the expression of P2X7 receptors in mouse ES cells that is coupled to an ionic current and mediates increased membrane permeability to Et^+^.

### 3.2 Exogenous ATP activates P2X7 receptor mediated necrotic cell death

**Fig. 4:**
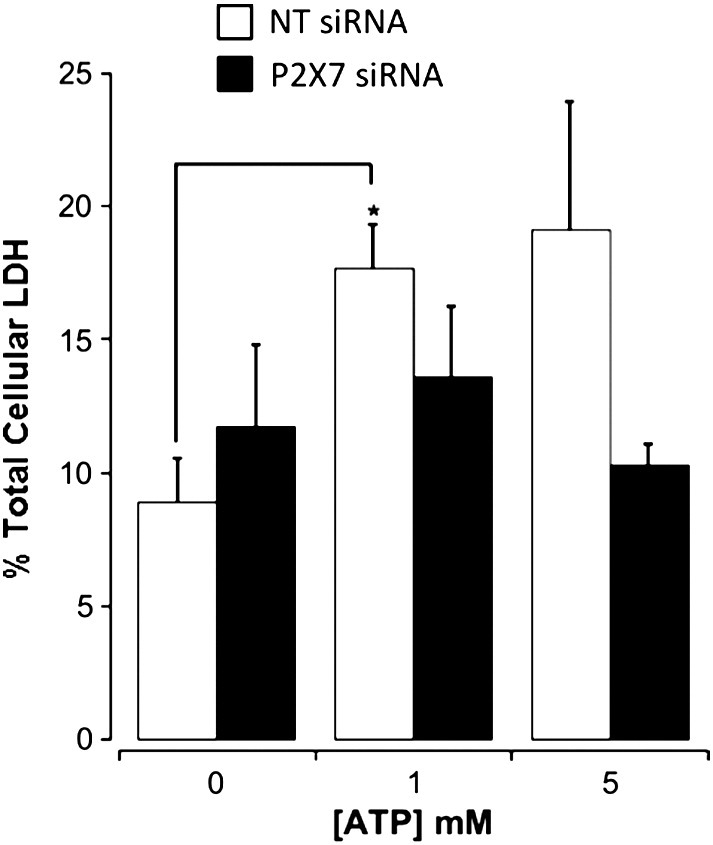
Stimulation of mouse ES cells with exogenous ATP leads to necrosis. Release of lactate dehydrogenase (LDH) as a function of total cellular LDH (plotted as % total cellular LDH) for mouse ES cells transfected with 20 nM non-targeting siRNA (NT siRNA) or P2X7-targeted siRNA (P2X7 siRNA) as indicated (*n* = 3). Cells stimulated with ATP for 45 min at 37 °C. * P < 0.05 by unpaired two-tailed t test.

**Fig. 5:**
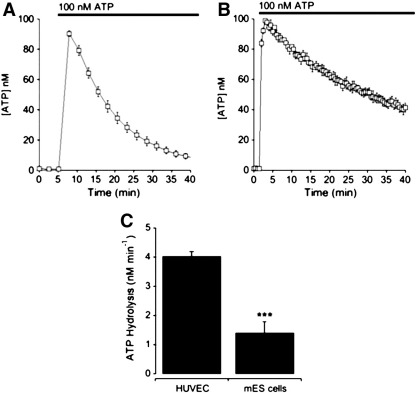
Comparison of exogenous ATP hydrolysis between mES cells and human umbilical vein endothelial cells. (A–B) Measurement of extracellular ATP levels following addition of 100 nM ATP for HUVEC (A) or mES cells (B). (C) Histogram comparing the rate of ATP metabolism for HUVEC (*n* = 4) versus mouse ES cells (*n* = 5). Data are plotted as the mean ± SEM. *** P < 0.001 by unpaired two-tailed t test.

In differentiated cells of the immune system, P2X7 activation is associated with rapid loss of cell viability by necrotic or apoptotic cell death [24,30,32–34]. We have investigated cell viability, and specifically necrotic cell death, upon exposure of mouse ES cells to exogenous ATP. Necrosis was detected by the release of lactate dehydrogenase (LDH) into the external medium as a function of total cellular LDH. Stimulation with exogenous ATP elicited the release of LDH following 45 min of agonist application (Fig. 4). Reduction of P2X7 receptor expression by siRNA resulted in an attenuation of ATP-mediated cell lysis demonstrating a role for P2X7 receptors (Fig. 4). Next we investigated the metabolism of exogenous ATP by ES cells compared to human umbilical vein endothelial cells (HUVECs) which have previously been demonstrated to have high NTP diphosphohydrolase CD39 activity [35]. HUVECs rapidly degraded exogenous ATP with an initial rate of 4.03 ± 0.26 nM min^− 1^ (*n* = 4) whilst mouse ES cells degraded ATP with an initial rate of 1.41 ± 0.37 nM min^− 1^ (*n* = 5) (Fig. 5A–C). These data demonstrate that exposure to high ATP concentrations leads to detectable necrotic cell death, and that mouse ES cells break down extracellular ATP more slowly than HUVECs. Although the ATP concentration within the environment surrounding the inner cell mass is unknown, this study demonstrates that mouse ES cells slowly metabolise exogenous ATP which maybe an important characteristic of these cells to facilitate the detection of excessive autocrine or paracrine ATP release.

### 3.3 Role of P2X7 receptors in ES cell proliferation, self-renewal and colony formation

Next we investigated whether *endogenous* ATP could activate P2X7 and whether this played an important functional role in the cell survival, self-renewal or proliferation of mouse ES cells. Previous studies have shown that *exogenous* ATP increases cell proliferation but did not alter alkaline phosphatase activity in the absence of serum [11]. Broad-spectrum P2 receptor inhibitors (suramin and reactive blue 2) reduced exogenous ATP elicited proliferation but did not alter basal proliferation in the presence of endogenous ATP [11]. In the current study, we measured basal extracellular ATP in the ES cell culture using the luciferase detection system as 0.54 ± 0.17 nM (*n* = 4). This is however a global measure of external ATP in the culture and does not reflect localised near-cell ATP levels that would be predicted to be substantially higher. To investigate the role of endogenous ATP and P2X7 activation in ES cell proliferation we used an MTS (3-(4,5-dimethylthiazol-2-yl)-5-(3-carboxymethoxyphenyl)-2-(4-sulfophenyl)-2H-tetrazolium) assay. In the presence of serum, it was found that P2X7 inhibition with A438079 or incubation with an ecto-ATPase (Apyrase) did not reduce cell proliferation (48 h: 10 μM A438079: 114 ± 2.9% control (*n* = 9) and 4 U ml^− 1^ Apyrase: 112 ± 3% control (*n* = 6)). These data demonstrate that endogenous ATP does not alter ES cell proliferation via P2X7 receptor activation.

**Fig. 6:**
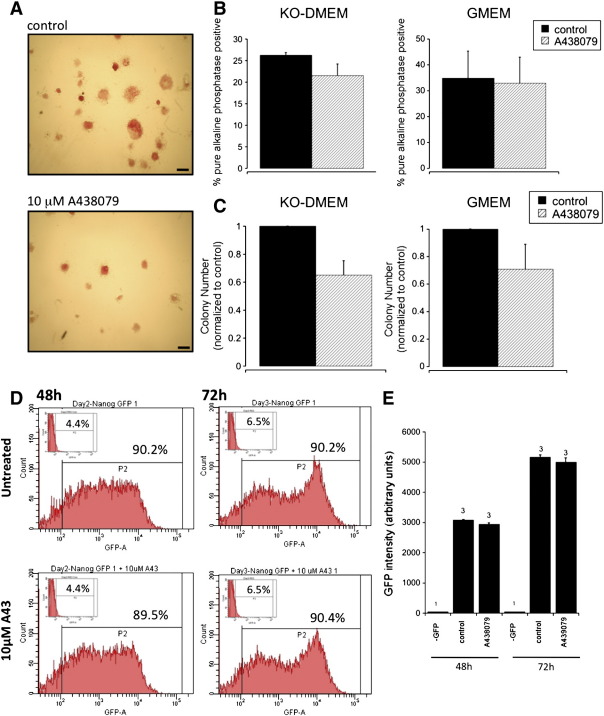
The role of P2X7 receptors in mouse ES cell colony number and self-renewal. Mouse ES cells were plated at approximately 166 cells/cm^2^ and cultured for 5 days (in the presence or absence of 10 μM A438079) prior to counting the percentage of colonies intensely staining for pure alkaline phosphatase activity (A–B) and total colony number normalised to control untreated cells (C). Cells cultured in GMEM or KO-DMEM (indicated in the figure) for 5 days as described in Materials and methods prior to analysis for colony number (C) and frequency of pure alkaline phosphatase positive colonies (B) where data are plotted as the mean ± SEM (*n* = 3). (A) A representative image of mES cells cultured in KO-DMEM in the presence or absence of 10 μM A438079. Scale bar = 400 μm. (D) Nanog-GFP expression in mES cells cultured in KO-DMEM for 48 and 72 h in the absence or presence of 10 μM A438079 as indicated in the figure. GFP fluorescence analysed by flow cytometry, representative images in figure with GFP^-ve^ control cells in insert. (E) Mean GFP fluorescence from flow cytometry experiments plotted as a histogram with *n* values indicated in the figure. Data plotted as mean ± SEM.

In ES cells, self-renewal is defined as proliferation accompanied by the suppression of differentiation to generate two identical daughter cells. To assess the role of P2X7 in self-renewal, ES cells were plated at a clonal density in the presence of LIF (and serum) in the presence or absence of the P2X7 antagonist, A438079. After 5 days, colonies were fixed and stained for alkaline phosphatase activity, expressed only by undifferentiated cells and hence a suitable marker of self-renewal [20]. Colonies that stained intensely for alkaline phosphatase and had a compact round morphology were defined as the ‘pure’ alkaline phosphatase positive colonies. Such colonies represent highly self-renewing ES cells. Our results demonstrate that treatment with 10 μM A438079 did not significantly alter the level of self-renewal compared to control cultures (*n* = 3, Fig. 6A and B). However, in the presence of the P2X7 antagonist A438079 (10 μM) the total colony number was reduced by 29 ± 17% (*n* = 3) in GMEM medium or 35 ± 10.2% (*n* = 3) in KO-DMEM compared to control untreated cells (Fig. 6C). Finally we have investigated the expression of a pluripotent cell specific transcription factor Nanog using mES cells expressing Nanog-green fluorescent protein (Nanog-GFP) [29]. It was found that Nanog-GFP expression was not altered by culture in the presence of 10 μM A438079 confirming that P2X7 inhibition does not alter ES cell self-renewal (Fig. 6D). In summary, P2X7 receptors do not regulate ES cell proliferation or self-renewal in response to endogenous ATP release but our data indicates a novel role in ES stem cell colony survival.

## 4 Discussion

Few studies have investigated the expression or functional role of P2X receptors during development, particularly in relation to P2X7 receptors. We have demonstrated the expression of P2X7 mRNA encoding several receptor isoforms in undifferentiated mouse ES cells and present data that support a key role of this receptor in ES cell survival. Several studies have investigated the expression of ion channels in ES cells and demonstrated the expression of GABA(A) receptors, voltage-gated calcium channels and potassium channels [36,37]. In mouse ES cells GABA signalling is reported to have differential actions on proliferation and differentiation according the receptor subtype activated. The discovery of ES cell expressed P2X7 receptors adds to the repertoire of ion channels that play an important role in controlling embryonic stem cell behaviour.

We have demonstrated that mouse ES cell P2X7 receptors couple to the opening of an ion channel followed by downstream membrane permeabilisation leading to the influx of Et^+^. A recent study has identified multiple splice variants of rat and mouse P2X7 receptors, where two splice variants differing in exon 1 have been studied in detail for rat and found to have distinct functional properties [38]. By RT-PCR, we have detected the expression of P2X7(k) mRNA. Homomeric rat P2X7(k) was found to have increased sensitivity to ATP, with currents activated at 10 μM ATP in contrast to 100 μM–1 mM ATP required to activate P2X7(a). In addition, the off-kinetics were strikingly different between P2X7(a) and P2X7(k) where a short agonist application evoked a current that reversed upon agonist removal for P2X7(a) whilst P2X7(k) currents were sustained upon agonist removal. Assuming comparable pharmacological properties for mouse P2X7(k) receptors then our biophysical data would suggest that homomeric P2X7(k) does not contribute to ES cell P2X7-mediated currents but this does not exclude a heteromeric receptor assembly that incorporates P2X7(k). Further studies are required to elucidate the variant(s) contribution to mouse ES cell P2X7 receptor-dependent responses.

In the adult organism, P2X7 is expressed in cells of the hematopoietic lineage, epithelium and endothelium [9]. The functional role of P2X7 receptors has been extensively characterised in immune cells where activation is associated with inflammatory responses and P2X7 acts as a cytotoxic receptor associated with apoptotic or necrotic cell death [24,39]. Indeed, chronic activation of ES cell P2X7 receptors by exogenous millimolar levels of ATP leads to detectable necrotic cell death within 1 h. However, there is also increasing evidence in the literature to support a role for P2X7 as a pro-survival receptor. Much of this data comes from studies using recombinant P2X7 receptors expressed in a HEK293 cell line, which leads to increased cell survival in serum-free conditions [16,17]. It is proposed that low levels of ATP in the extracellular milieu lead to the basal activation of P2X7 receptors that couple to signalling pathways to promote cell survival [16–18]. Increased expression of P2X7 is proposed to contribute to the altered growth patterns and consequently the pathology of several cancers including chronic lymphocytic leukemia [15,19]. In mouse ES cells, we found that a selective P2X7 antagonist reduced their plating efficiency, resulting in formation of fewer colonies, suggesting that functional P2X7 receptors are required for optimal in vitro survival of mouse ES cells. This would fit with the current model that P2X7 can function both as a pro-survival or cytotoxic receptor dependent on the mode of receptor activation. The current model for P2X7 leading to altered cell survival and/or proliferation is primarily based on altered intracellular Ca^2 +^ signalling. P2X7 receptor expression is reported to lead to an increase in mitochondrial and endoplasmic reticulum Ca^2+^ content, changes in cellular ATP levels and activation of nuclear factor of activated T cells complex 1 (NFATc1), that protects from apoptotic cell death from other stimuli [16–18]. We hypothesise that P2X7-dependent changes in intracellular Ca^2+^ are an important regulator of mouse ES cell colony survival. Cytosolic Ca^2+^ signalling has been shown to play an important role in embryonic development [40]. Indeed, one study has demonstrated cell cycle-dependent Ca^2+^ oscillations are required for G1/S progression of mouse embryonic stem cells [41]. However, the role for P2X7 in optimal mouse ES cell survival in vitro would suggest that P2X7 knockout mice might show embryonic lethality. The fact that three different P2X7 knockout mice have now been successfully generated might suggest that the in vivo role of P2X7 is not as important in survival as seen in vitro or the incomplete block of stem cell survival is not sufficient to alter embryo survival [42–44]. To further understand the potential role of P2X7 and ATP signalling in development, it would be fundamental to measure intra-blastocyst ATP levels and pericellular ATP levels at the inner cell mass. These are technically challenging experiments, however, technologies are developing to measure extracellular peri-cellular ATP in vivo and this would be an interesting direction of future research.

Finally, in differentiated cells of the immune system, P2X7 receptor activation is classically associated with necrotic or apoptotic cell death. Extracellular ATP is described as a ‘danger-associated molecular pattern’ linked with inflammatory signalling and cell death. In the current study, we provide evidence for coupling of P2X7 receptor stimulation to cell necrosis. These data, coupled with the expression of P2X7 receptors in mouse ES cells, suggest that receptor activity may be involved in basal cell survival in the inner cell mass as well as providing a mechanism to detect gross stress leading to excessive ATP secretion within the blastocyst. It would therefore be important to understand the expression profile of P2X7 receptors during differentiation to an array of progenitor cells that may be used for cell replacement therapies, as transplantation of ES cell-derived cells expressing P2X7 may have adverse consequences to cell survival in material to be used for cellular therapy and in transplanted tissues. Indeed, P2X7 expression has been demonstrated in mouse neural progenitor cells where receptor activation is associated with necrotic cell death [45].

## 5 Conclusions

Together these data demonstrate a previously unreported role for P2X7 receptors in control of ES cell biology, where these receptors can mediate a pro-survival or pro-death signal depending on the mode of activation.

## Figures and Tables

**Table 1:** Oligonucleotide primers.

Ensembl transcript ID	Primer names		Sequence 5′-3′	Annealing temp (°C)	Product size	Isoforms
ENSMUST00000100737ENSMUST00000031425ENSMUST00000121489ENSMUST00000086247	PanP2X7	F	GCTGGAACGATGTCTTGC	56	292 bp	A, B, C, D
		R	AAGAATGAGTTCCCCTGC			
ENSMUST00000100737	P2X7 A	F	GCCAAGAAGTTCCAAGACC	59	292 bp	A, K
		R	AGGGTGTCTCGGGACAGC			
ENSMUST00000121489	P2X7 C	P2X7B F	GTTTTCGGCACTGGAGGA	51	317 bp	C
		R	TAGGTTGGAACTTCTTGG			
ENSMUST00000031425	P2X7 B	F	GTTTTCGGCACTGGAGGA	54	309 bp	B
		R	TCAGGTCGCCATACATACAT			
ENSMUST00000086247	P2X7 D	Pan P2X7 F	GCTGGAACGATGTCTTGC	55	360 bp	D
		R	GGTTGGAAAGATCCTCAG			
ENSMUST00000167557	P2X7 K	F	CTGCCCGTGAGCCACTTAT	56	303 bp	K
		PanP2X7 R	AAGAATGAGTTCCCCTGC			

## References

[1] Evans M.J., Kaufman M.H. (1981). Nature.

[2] Martin G.R. (1981). Proceedings of the National Academy of Sciences of the United States of America.

[3] Eckfeldt C.E., Mendenhall E.M., Verfaillie C.M. (2005). Nature Reviews Molecular Cell Biology.

[4] Boeuf H., Hauss C., Graeve F.D., Baran N., Kedinger C. (1997). The Journal of Cell Biology.

[5] Cartwright P., McLean C., Sheppard A., Rivett D., Jones K., Dalton S. (2005). Development.

[6] Niwa H., Burdon T., Chambers I., Smith A. (1998). Genes & Development.

[7] Ying Q.L., Nichols J., Chambers I., Smith A. (2003). Cell.

[8] Sato N., Meijer L., Skaltsounis L., Greengard P., Brivanlou A.H. (2004). Nature Medicine.

[9] North R.A. (2002). Physiological Reviews.

[10] Adinolfi E., Pizzirani C., Idzko M., Panther E., Norgauer J., Di Virgilio F., Ferrari D. (2005). Purinergic Signal.

[11] Heo J.S., Han H.J. (2006). Stem Cells.

[12] Khaira S.K., Pouton C.W., Haynes J.M. (2009). British Journal of Pharmacology.

[13] Storm M.P., Kumpfmueller B., Thompson B., Kolde R., Vilo J., Hummel O., Schulz H., Welham M.J. (2009). Stem Cells.

[14] Surprenant A., Rassendren F., Kawashima E., North R.A., Buell G. (1996). Science.

[15] Adinolfi E., Melchiorri L., Falzoni S., Chiozzi P., Morelli A., Tieghi A., Cuneo A., Castoldi G., Di Virgilio F., Baricordi O.R. (2002). Blood.

[16] Adinolfi E., Callegari M.G., Ferrari D., Bolognesi C., Minelli M., Wieckowski M.R., Pinton P., Rizzuto R., Di Virgilio F. (2005). Molecular Biology of the Cell.

[17] Adinolfi E., Callegari M.G., Cirillo M., Pinton P., Giorgi C., Cavagna D., Rizzuto R., Di Virgilio F. (2009). Journal of Biological Chemistry.

[18] Adinolfi E., Callegari M.G., Woltersdorf R., Falzoni S., Chiozzi P., Pellegatti P., Callegari M.G., Sandonà D., Markwardt F., Schmalzing G., Di Virgilio F. (2010). The FASEB Journal.

[19] Baricordi O.R., Melchiorri L., Adinolfi E., Falzoni S., Chiozzi P., Buell G., Di Virgilio F. (1999). Journal of Biological Chemistry.

[20] Paling N.R., Wheadon H., Bone H.K. (2004). Journal of Biological Chemistry.

[21] Paling N.R., Welham M.J. (2005). Blood.

[22] Jaffe E.A., Nachman R.L., Becker C.G., Minick C.R. (1973). The Journal of Clinical Investigation.

[23] Storm M., Bone H.K., Beck C.G. (2007). Journal of Biological Chemistry.

[24] Moore S.F., MacKenzie A.B. (2009). Journal of Immunology.

[25] Hewinson J., Moore S.F., Glover C., Watts A.G., MacKenzie A.B. (2008). Journal of Immunology.

[26] Donnelly-Roberts D.L., Namovic M.T., Faltynek C.R., Jarvis M.F. (2004). Journal of Pharmacology and Experimental Therapeutics.

[27] Jiang L.H., Rassendren F., Mackenzie A., Zhang Y.H., Surprenant A., North R.A. (2005). American Journal of Physiology. Cell Physiology.

[28] Beigi B.D., Dubyak G.R. (2000). Journal of Immunology.

[29] Chambers I., Silva J., Colby D., Nichols J., Robertson M., Nijmeijer B., Vrana J., Jones K., Grotewold L., Smith A. (2007). Nature.

[30] Moore S.F., MacKenzie A.B. (2007). Cellular Signalling.

[31] Donnelly-Roberts D.L., Namovic M.T., Han P., Jarvis M.F. (2009). British Journal of Pharmacology.

[32] Ferrari D., Chiozzi P., Falzoni S., Dal Susino M., Collo G., Buell G., Di F. (1997). Virgilio.

[33] Taylor S.R., Gonzalez-Begne M., Dewhurst S., Chimini G., Higgins C.F., Melvin J.E., Elliott J.I. (2008). Journal of Immunology.

[34] Tsukimoto M., Maehata M., Harada H., Ikari A., Takagi K., Degawa M. (2006). Journal of Immunology.

[35] Marcus A.J., Broekman M.J., Drosopoulos J.H., Islam N., Alyonycheva T.N., Safier L.B., Hajjar K.A., Posnett D.N., Schoenborn M.A., Schooley K.A., Gayle R.B., Maliszewski C.R. (1997). The Journal of Clinical Investigation.

[36] Schwirtlich M., Emri Z., Antal K., Máté Z., Katarova Z., Szabó G. (2010). The FASEB Journal.

[37] Ng S.Y., Chin C.H., Lau Y.T., Luo J., Wong C.K., Bian Z.X., Tsang S.Y. (2010). Journal of Cellular Physiology.

[38] Nicke A., Kuan K.H., Masin M., Rettinger J., Marquez-Klaka B., Bender O., Górecki D.C., Murrell-Lagnado R.D., Soto F. (2009). Journal of Biological Chemistry.

[39] Di Virgilio F. (2007). Trends in Pharmacological Sciences.

[40] Whitaker M. (2006). Physiological Reviews.

[41] Kapur N., Mignery G.A., Banach K. (2007). American Journal of Physiology. Cell Physiology.

[42] Chessell I.P., Hatcher J.P., Bountra C., Michel A.D., Hughes J.P., Green P., Egerton J., Murfin M., Richardson J., Peck W.L., Grahames C.B., Casula M.A., Yiangou Y., Birch R., Anand P., Buell G.N. (2005). Pain.

[43] Solle M., Labasi J., Perregaux D.G., Stam E., Petrushova N., Koller B.H., Griffiths R.J., Gabel C.A. (2001). Journal of Biological Chemistry.

[44] Qu Y., Misaghi S., Newton K., Gilmour L.L., Louie S., Cupp J.E., Dubyak G.R., Hackos D., Dixit V.M. (2011). Journal of Immunology.

[45] Delarasse C., Gonnord P., Galante M., Auger R., Daniel H., Motta I., Kanellopoulos J.M. (2009). Journal of Neurochemistry.

